# Degradation of Microcystin-LR and RR by a *Stenotrophomonas* sp. Strain EMS Isolated from Lake Taihu, China

**DOI:** 10.3390/ijms11030896

**Published:** 2010-03-02

**Authors:** Jian Chen, Liang Bin Hu, Wei Zhou, Shao Hua Yan, Jing Dong Yang, Yan Feng Xue, Zhi Qi Shi

**Affiliations:** 1 Institute of Food Safety and Quality, Jiangsu Academy of Agricultural Sciences, 50 Zhongling Street, Nanjing 210014, China; E-Mails: chenjian@jaas.ac.cn (J.C.); zhouwei@jaas.ac.cn (W.Z.); smartforest@yahoo.cn (J.D.Y.); xueyanfeng@jaas.ac.cn (Y.F.X.); 2 School of Food, Henan Institute of Science and Technology, Xinxiang 453003, China; E-Mail: hlb197988@163.com (L.B.H.); 3 Key Laboratory of Food Safety Monitoring and Management, Ministry of Agriculture, 50 Zhongling Street, Nanjing 210014, China; E-Mail: shyan@jaas.ac.cn (S.H.Y.); 4 Institute of Resources and Environmental Sciences, Jiangsu Academy of Agricultural Sciences, 50 Zhongling Street, Nanjing 210014, China

**Keywords:** degradation, microcystin-LR, microcystin-RR, cyanobacteria, EMS, *mlrA*

## Abstract

A bacterial strain EMS with the capability of degrading microcystins (MCs) was isolated from Lake Taihu, China. The bacterium was tentatively identified as a *Stenotrophomonas* sp. The bacterium could completely consume MC-LR and MC-RR within 24 hours at a concentration of 0.7 μg/mL and 1.7 μg/mL, respectively. The degradation of MC-LR and MC-RR by EMS occurred preferentially in an alkaline environment. In addition, *mlrA* gene involved in the degradation of MC-LR and MC-RR was detected in EMS. Due to the limited literature this gene has rare homologues. Sequencing analysis of the translated protein from *mlrA* suggested that MlrA might be a transmembrane protein, which suggests a possible new protease family having unique function.

## 1. Introduction

In recent years, toxic cyanobacterial blooms have occurred frequently in eutrophic lakes, rivers and reservoirs in China [1–3]. Besides the damage to the aquatic ecosystem, toxin production is another extremely dangerous consequence of cyanobacterial blooms. The most commonly occurring toxins produced by these cyanobacteria are microcystins (MCs), which are cyclic heptapeptides and share common structural feature, including the Adda (3-amino-9-methoxy-2,6,8-trimethyl-10-phenyl-4,6-decadienoic acid) side chain and a ring, consisting of five amino acids. More than 90 isoforms of microcystin have been discovered [4]. The variations occur between different strains of the same genus, as well as between different genera. In China, cyanobacterial blooms are common symptoms of water eutrophication caused by agricultural and industrial pollution. Microcystin-LR (MC-LR) and microcystin-RR (MC-RR) are the most frequent MC variants, which pose a grave threat to both environmental safety and public health [5]. MCs are extremely toxic to human and animals by causing the damage of DNA, inhibiting the activities of phosphatases 1 and 2A, and even triggering the enhanced transcriptional level of proto-oncogene in liver [6–11]. In addition, abundant literature has described the phytotoxic symptoms of microcystins, such as chlorophyll reduction, oxidative stress, histological damage, and growth inhibition, etc. [12–16].

Lake Taihu is the third largest fresh water lake in China. Due to the increasingly sharp water eutrophication in recent years, cyanobacteiral blooms occur frequently in Lake Taihu. [17–19]. The annual distribution area of cyanobacterial blooms in Lake Taihu remained relatively constant at a level of approximately 62.2 km^2^ for 1987–2000, but the cyanobacterial bloom area has shown a significant increase after 2000. In 2007, it even reached 979.1 km^2^, which was almost 2/5 of the total area of Lake Taihu [20]. Although water management has been performed, water eutrophication and cyanobacterial blooms are not significantly controlled due to the massive discharge of industrial wastewaters into Lake Taihu. A guideline value of 1.0 μg/L for MC-LR in drinking water had been set by Ministry of Health of the People’s Republic of China according to the guideline of the World Health Organization [21,22]. Bioremediation of microcystin-contaminated water bodies involves the utilization of natural microorganisms to degrade MCs. At present, bioremediation is accepted because it is cost-effective; efficiently removes microcystins, and protects Nature [23]. The construction of biofilters with MCs-degrading bacteria showed the feasibility of bioremediating MCs-contaminated water using bacteria [24,25]. To date, only a small number of bacteria with the ability to degrade MCs have been isolated. These bacteria genera mainly belong to *Proteobacteria*, such as *Sphingomonas*, *Paucibacter*, *Sphingopyxis*, *Burkholderia*, and *Methylobacillus* [16,26–32]. In 2009, Manage *et al*. found three new microcystin degraders that did not belong to *Proteobacteria* [33].

To date, the only reported mechanism of MC biodegradation was investigated by Bourne *et al.* [27] in *Sphingomonas*. It was a step-by-step pathway mediated by three enzymes named MlrA, MlrB, and MlrC, respectively. Bourne *et al.* [34] had characterized the gene cluster including *mlrA*, *mlrB*, *mlrC* in *Sphingomonas* sp.. MlrA (microcystinase) coding by *mlrA* gene breaks off the cyclic peptide of MC-LR, which is the first and most important step in this pathway. The product from MlrA-catalyzed process is a linear peptide, which shows much lower toxicity than MC-LR. However, *mlrA* has very limited homologues in the DNA databases according to the literature [28,34].

The key to bioremediate MCs-contaminated water is to employ native bacteria in order to avoid biotic intrusion. Limited native microorganisms with high efficiency of MCs degradation have been reported in China [26,35,36]. Therefore, the objectives of the present study were to: (1) isolate single bacterial strains with strong MC-degrading ability from the sludge of piled cyanobacteria fished out from Lake Taihu; (2) identify the strain(s) based on physical and chemical characterization as well as 16S rRNA gene sequencing; (3) explore the optimal condition of MCs degradation by the strain(s); (4) investigate the mechanism of bacterial degradation of MCs by detecting *mlrA* gene from the identified bacterial strains.

## 2. Results and Discussion

### 2.1. Results

#### 2.1.1. Isolation and Identification of MC-Degrading Bacteria EMS

**Figure 1.**
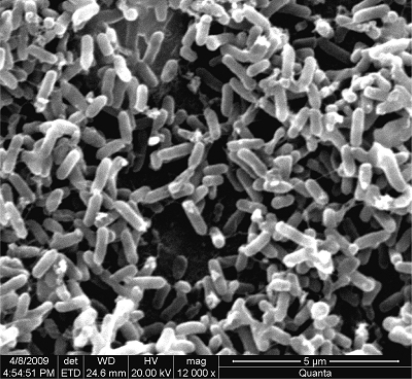
Scanning electron microscopy image of EMS cells.

In order to obtain native bacteria with high MC-degradation efficiency, a cyanobacterial sludge sample from blue-green algae piled in the Lake Taihu region was collected and screened according to the method described in Section 3.3. Fifteen isolated single colonies were inoculated into the aqueous MS medium containing crude MC extract. Among the test strains, only the EMS strain showed the ability to degrade MCs. The EMS strain could completely degrade the added MCs within 24 h. This strain was aerobic, gram-negative and rod-shaped. The cells were 1.6 ± 0.6 μm in length and 0.4 ± 0.1 μm in width, immotile, and produced white round colonies on MS agar medium. No spores were observed under microscope (Figure 1).

**Figure 2.**
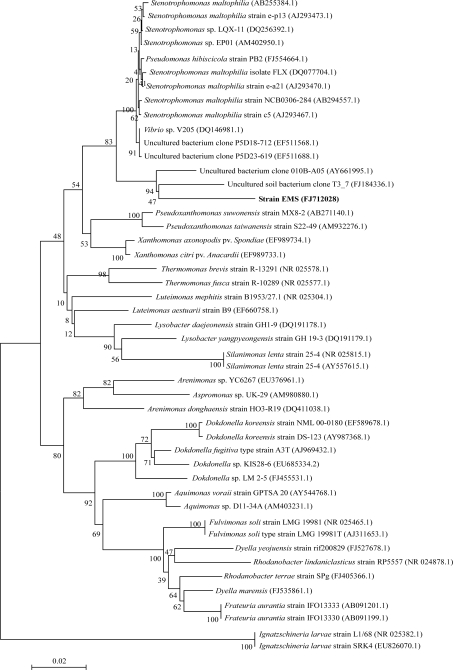
Phylogenetic tree indicating the position of strain EMS in the established related species of the genus. The value on each branch is the estimated confidence limit (expressed as a percentage) for the position of the branch as determined by a bootstrap analysis.

The online BLAST search showed that among the sequences of the established species the 16S rRNA gene sequence of the EMS strain was most similar to the sequence of *Stenotrophomonas maltophilia* (94% similarity). Evolutionary distances were calculated for a dataset that consisted of the sequences of the EMS strain and 46 other species of *Xanthomonadaceae*, including *Stenotrophomonas* sp. and some uncultured bacteria. A neighbor-joining phylogenetic tree was reconstructed on the basis of the obtained distance matrix data (Figure 2). The EMS strain (Genbank accession number: FJ712028) was included in the big cluster of genus *Stenotrophomonas*, *Pseudoxanthomonas* and *Xanthomonas*. Although EMS was close to *Stenotrophomonas* sp. according to the evolutionary distance by chemotaxonomic analysis, it constituted an independent cluster with another uncultured bacterium (FJ184336) (Figure 2), which had been submitted to NCBI as capable of degrading pyrene. Therefore, the identification of EMS strain in species division needs to be performed by DNA-DNA hybridization and intensive phenotype characterization.

#### 2.1.2. Biodegradation of MC-LR and MC-RR by Strain EMS

**Figure 3.**
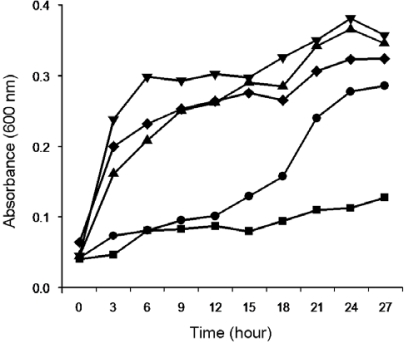
The effect of different temperatures on EMS growth indicated by absorbance at 600 nm measured every 3 hours. EMS was incubated with M9 medium containing crude MCs extract at 4 °C (▪), 10 °C (•), 20 °C (▴), 30 °C (▾), and 37 °C (♦), respectively.

**Figure 4.**
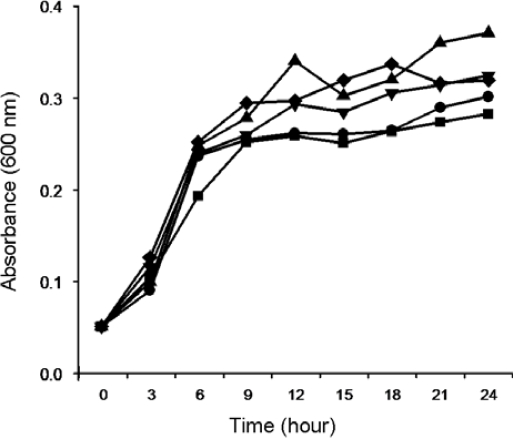
The effect of different pH values on EMS growth indicated by absorbance at 600 nm measured every 3 hours. EMS was incubated with M9 medium containing crude MCs extract with pH values at 5.0 (▪), 6.0 (•), 7.0 (▴), 8.0 (▾), and 9.0 (♦), respectively.

Firstly, we examined the impact of temperature and pH on the growth of EMS strain in medium containing crude MC extract. Temperature significantly affected the growth of EMS. Among the tested temperatures (4–37 °C) EMS strain showed the highest growth rate between 30 °C and 37 °C (Figure 3), but the growth of EMS strain did not show any significant difference in mediums with different pH values (5.0–9.0) (Figure 4). Therefore, the degradation of MC-LR and MC-RR by EMS were tested at an incubation temperature at 30 °C under different pH values in order to identify the effects of pH on the EMS-mediated degradation of MC-LR and MC-RR.

**Figure 5.**
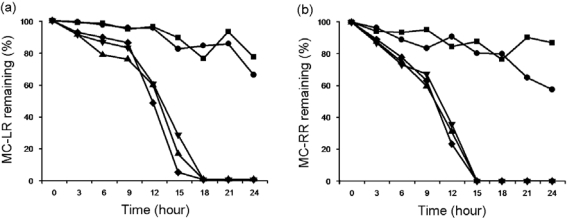
The effect of different incubation pH values on the degradation of MC-LR (a) and MC-RR (b) by strain EMS. EMS was incubated with M9 medium containing crude MCs extract with pH values pH values at 5.0 (▪), 6.0 (•), 7.0 (▴), 8.0 (▾), and 9.0 (♦), respectively. The incubation temperature was 30 °C. Initial concentration of MC-LR and MC-RR in medium was 1.53 μg/mL and 0.82 μg/mL, respectively.

EMS preferred to degrade MC-LR and MC-RR in alkaline rather than acidic environments. After incubation for 24 h, EMS consumed 22% and 34% of MC-LR in cultures at pH 5.0 and 6.0, respectively, but MC-LR was completely degraded within 18 h at pH 7.0 to 9.0 (Figure 5a). Similarly, EMS consumed 11% and 41% of MC-RR within 24 h in culture at pH 5.0 and 6.0, respectively, but MC-RR was completely degraded within 15 h at pH at 7.0 to 9.0 (Figure 5b).

**Figure 6.**
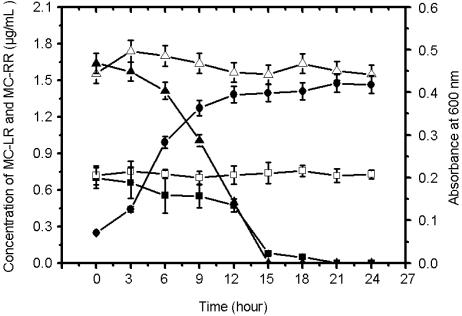
Degradation of MC-LR and MC-RR by strain EMS. Strain EMS growth in M9 medium containing crude MCs extract was detected by measuring the absorbance of the culture at 600 nm (•). Concentrations of MC-LR (▪) and MC-RR (▴) were measured every 3 hours. ( ) and (□) indicate the concentrations of MC-LR and MC-RR in medium without bacteria, respectively. Vertical bars represent standard deviation of the mean (n = 3).

Next, we tested the degradation of MC-RR and MC-LR by EMS in culture under the optimal conditions of pH 7.0 at 30 °C. The initial concentration of MC-RR and MC-LR in culture was 1.7 μg/mL and 0.7 μg/mL, respectively. Compared to the controls, both MC-LR and MC-RR had been completely degraded by the EMS strain within 24 h. The decrease in the concentration of MC-LR and MC-RR were accompanied by the increase in bacterial growth, as measured by absorbance at 600 nm (Figure 6).

**Figure 7.**
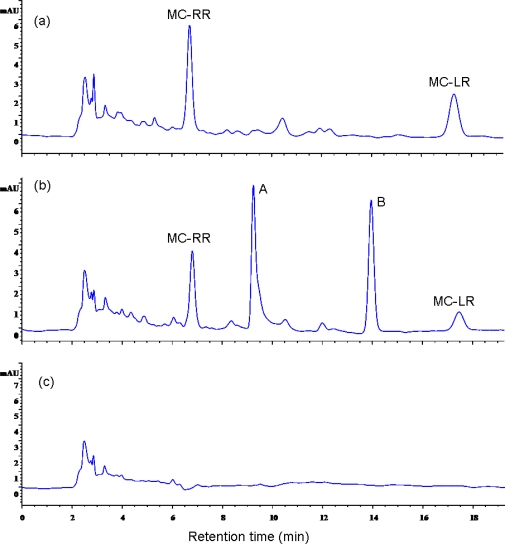
HPLC chromatograms at time zero (a), 12 h (b), and 24 h (c), illustrating degradation of MC-LR and MC-RR incubated with EMS.

HPLC chromatograms revealed the retention time of MC-RR and MC-LR was 7.0 min and 17.5 min, respectively (Figure 7a). The area of two peaks of both MC-RR and MC-LR decreased significantly after incubation for 12 h than those of in the very beginning (Figure 7b). There were two intermediate products that could be clearly seen as separate peaks A and B in time sequence HPLC chromatograms as well (Figure 7b). The disappearance of all peaks indicated the complete degradation of MC-LR and MC-RR by EMS after incubation for 24 h, as shown in Figure 7c.

#### 2.1.3. Detection of mlrA Gene from MCs-degrading Bacteria Strain EMS

**Figure 8.**
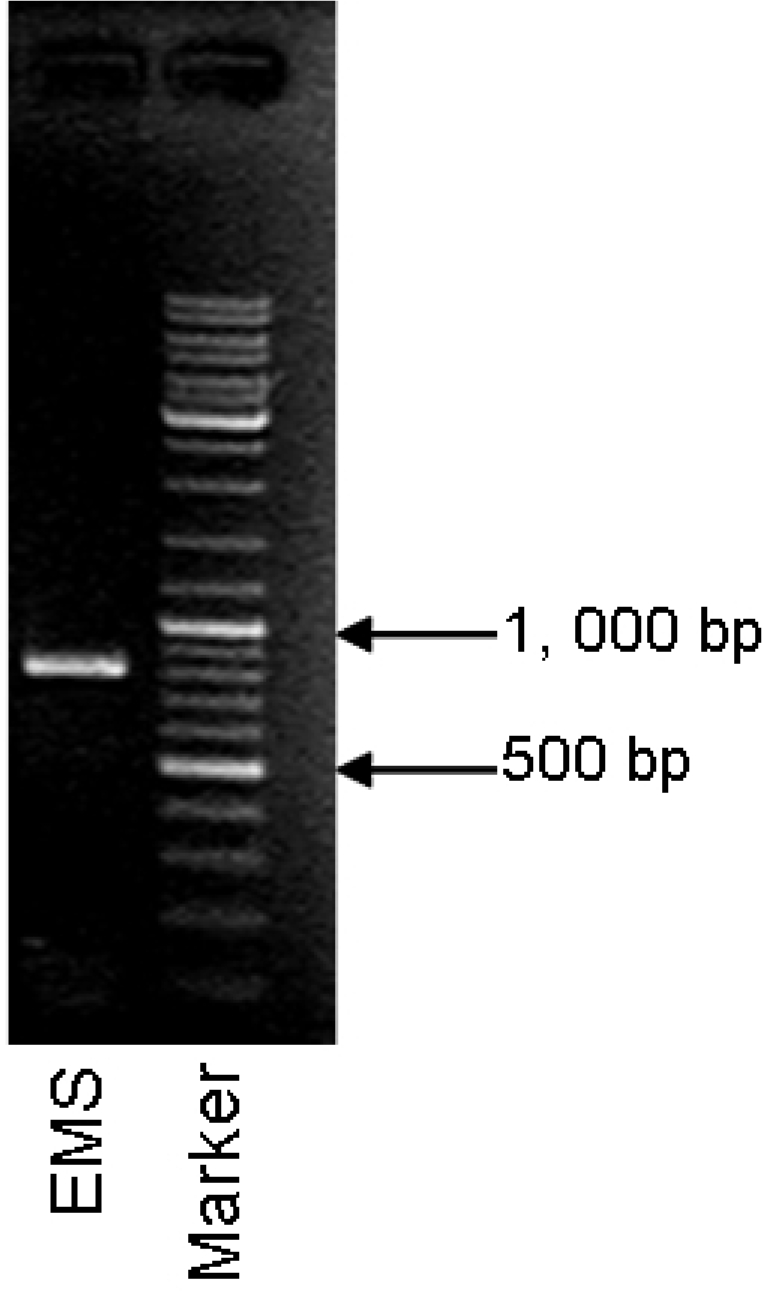
Detection of *mlrA* gene fragment by PCR in MCs-degrading strain EMS.

Sense and antisense primers for *mlrA* amplification were located on the positions 103–123 and 891–911 on *mlrA* gene of the ACM-3962 strain according to the reports of Bourne *et al.* [34] and Satio *et al.* [28]. A single band with the expected size (801 bp) was detected by PCR (Figure 8). An online search in GeneBank suggested that only five *mlrA* homologs had been reported in five bacterial strains. Two of them belong to *Sphingopyxis* sp. while the others belong to *Sphingomonas* sp. The GeneBank accession numbers of these *mlrA* homologus are AB468058, DQ112243, AF411068, AB114202, and AB114203. Our amplified products from EMS showed high similarity (83%–98%) with *mlrA* gene sequences that had been reported in the literature (Table 1), therefore, we concluded that the EMS strain contained a *mlrA* homologue, which was temporally named *mlrA-EMS* in this study.

**Figure 9.**
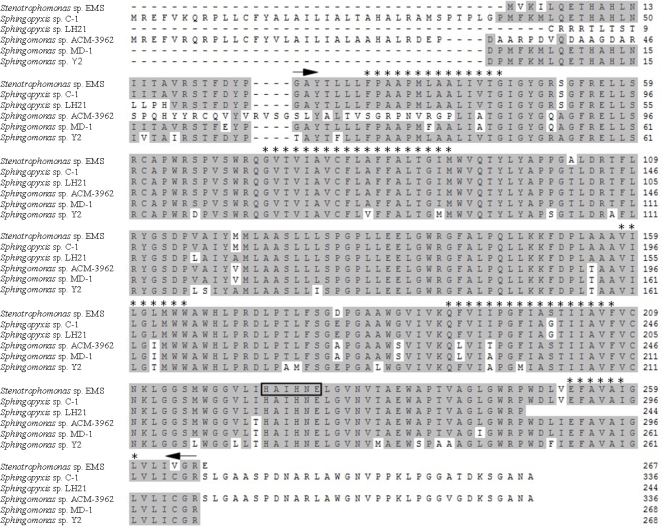
Alignment of the amino acid sequence of MlrA-EMS with other putative MlrAs. Grey sections indicate the identical residues in the compared sequences. The region between two black arrows is the highly conserved region in MlrA. Black frame indicates a classic zinc-bonding site. And * indicate the predicted transmembrane region with alpha helix.

The putative protein named as MlrA-EMS translated from *mlrA-EMS* gene was constituted by 267 amino acids. The multialignment of MlrA-EMS with five other MlrA sequences was performed by MegAlign in DNAStar (DNASTAR, Inc., Wisconsin, USA). MlrA-EMS showed high amino acid sequence similarities with other MlrAs. A highly conserved region was located between G^26^ to R^266^ in MlrA-EMS (Figure 9). ProtParam analysis suggested that MlrA-EMS might be a hydrophobic protein. Five alpha helixes (indicated as * in Figure 9) that were considered to be transmembrane regions were found in MlrA as well.

### 2.2. Discussion

Bioremediation has been developed as a new technique of remediating MC-contaminated water. Most of the MC-degrading bacteria that had been identified until now were preoteobacteria, as reviewed by Edwards and Lawton [23]. In the present study, a EMS strain with a high efficiency for degrading MC-LR and MC-RR was isolated from a cyanobacterial sludge from the Lake Taihu region, China. EMS was tentatively identified as a member of the *Stenotrophomonas* genus that belongs to *Xanthomonadaceae*, which is one of the subdivisions of the gamma division of proteobacteria containing many microorganisms with the capability of degrading harmful substances [37,38]. Satio *et al.* [39] and Jones *et al.* [40] had reported that the indigenous bacteria did not require a lag time to commence microcystin degradation after acclimatization with microcystin LR. In our study, EMS strain was isolated from mixed bacteria after acclimatization with MCs. Finally, there was no lag time for the growth of EMS strain in culture containing MCs, which revealed that acclimatization made EMS produce specific enzyme(s) to degrade MCs. This suggests that EMS became the dominant strain in mixed bacteria after succession. The aquatic environment became alkaline during bloom decay or bloom lysis with rapid collapse of cyanobacterial populations [41]. Our results showed that the degradation of MCs by strain EMS was significantly affected by different pH values of the incubation culture. The alkaline preference for MC degradation by EMS indicated that the MC-degrading enzyme (in EMS might be an alkaline protease, which agrees well with the report that MC-LR could be degraded by alkaline protease secreted by *Pseudomonas aeruginosa* [42].

Studies on the mechanisms of MC biodegradation are very limited because MCs are cyclic peptides, which are stable against general protease [28]. Although several bacterial genus with the capability of degrading MCs had been reported by different research groups [16,26–28,31,32,39,43,44], the only reported mechanism of MC-LR biodegradation was the three-enzymatic pathway in *Sphingomonas* sp. (strain ACM-3962) discovered through the detection and identification of intermediate degradation products [27,34]. Three enzymes (MlrA, MlrB, and MlrC) acted sequentially to degrade MC-LR. MlrA was involved in the first step during the biodegradation of MCs. We found two intermediate products after incubation of strain EMS with MCs for 12 h. And the detection of *mlrA* gene in EMS confirmed the similarity of MCs-degrading mechanism between EMS strain and *Sphingomonas* sp. (ACM-3962) [27,34]. MC-LR and MC-RR have the general structure cyclo-(Ala-*X*-MeAsp-Arg-Adda-Glu-Mdha), where *X* represents l-Leu and l-Arg in MC-LR and MC-RR, respectively [45]. MlrA was supposed to hydrolyze the peptide bond formed between Arg and Adda in MC-LR [27]. Therefore, MlrA in EMS probably involved in the first step of hydrolysis of both MC-LR and MC-RR, both of which contained MlrA-acting site (-Arg-Adda-). The cyclic structure is the most toxic part in MC molecules. The products from the hydrolysis catalyzed by MlrA showed no toxicity to animals because MlrA acted by breaking the ring in MCs. Therefore, MlrA is the key enzyme during the biodegradation of MCs [27]. Except for the detection of *mlrA* gene in *Sphingomonas* genus (AF411068, AB114202, and AB114203) [28,34] and *Sphingopyxis* genus (AB468058, DQ112243), both of which belonged to alpha-proteobacteria, our present study is the first report of *mlrA* gene detection in *Stenotrophomonas* genus that belonged to the gamma division of proteobacteria. Multialignment analysis suggested that *mlrA* gene is highly conserved in three different bacterial genuses, so it can be concluded that *mlrA* gene is probably highly relevant to microcystin degradation but not unique to any bacterial genus.

There is very little information about the protein MlrA due to the limited number of homologues and similarities in the literature. Bourn *et al.* [34] tentatively identified MlrA as a metalloprotease-like enzyme because of the inhibition of MlrA activity by metalloprotease inhibitors EDTA and 1,10-phenanthroline, as well as the prediction of a of classic zinc-binding motif (HEXXH) that was found to be a signature representative of metalloprotease. MlrA-EMS contained the same motif (H^225^AIH^228^NE^230^ H^223^) in its sequence as well. A highly conserved region located between G^26^ to R^266^ was found based on the multialignemnt of all the reported MlrA-like fragments. In addition to being a homologue of the CAAX amino-terminal protease family with unknown functions, as predicted by Satio *et al.* [28], we also performed the analysis of the transmembrane region and the hydropathicity of MlrA. MlrA might be a transmembrane protein as suggested by the discoveries of strong hydropathicity and five transmembrane regions in the conserved region. Subcellular location analysis indicated that MlrA was probably located in the plasma membrane. Some metalloproteases have been located on membrane and are involved in variable physiological process as receptor of signals of a signal peptide [46,47]. Bourne *et al.* [34] predicted a signal peptide with the cleavage site between the alanine and leucine amino acids at positions 26 and 27 in MlrA, respectively. Our previous study suggested that the fragments of cell membrane and cell wall showed biodegradative activity of both MC-LR and MC-RR (data not shown). Therefore, MlrA possibly mediated the process of MC-LR degradation with a possible location in the plasma membrane. However, these predictions need to be verified by X-ray crystallography, site-directed mutagenesis, and co-immunoprecipitation experiments in order to characterize MlrA and illuminate its function specifically.

## 3. Experimental Section

### 3.1. Extraction of MCs

Fresh cyanobacteria were fished out from Lake Taihu in May, 2007 when extensive and serious cyanobacterial blooms occurred. Fresh cyanobacteria were dehydrated by lyophilization. About 0.2 g of cyanobacterial cells were mixed with 10 mL methanol-water (80:20, v: v) followed by sonication for 1 h. Then the mixture was centrifuged at 12,000 rev/min for 10 min at 4 °C. The supernatant was concentrated by rotary evaporation at 40 °C to remove methanol. The resulting extract was diluted to 5 mL with distilled water and the pH adjusted to 3.0. Then the extract was centrifuged at 12,000 rev/min for 10 min at 4 °C followed by through a 0.2 μm cut-off Acrodisc syringe^®^ filter (Pall Corporation, Saint-Germain-en-Laye, France). Subsequently, the pH value of extract was adjusted to 7.0. After being autoclaved, the extract was diluted to 5 mL with sterilized water and stored at −20 °C before use in the following experiment. Toxin analysis of the crude extract revealed the concentration of MC-RR and MC-LR at 18 μg/mL and 8 μg/mL, respectively.

### 3.2. Detection and Quantification of MC-LR and MC-RR

Detection and quantification of toxins were performed using high-performance liquid chromatography (HPLC). Briefly, the crude extract was thawed in room temperature and centrifuged at 12,000 rev/min for 10 min at 4 °C. The supernatant were applied to a conditioned SPE cartridge (SepPak C18, Waters). The cartridge was firstly washed with 5 mL methanol followed by 5 mL distilled water. Impurities were eluted with 2 mL methanol and MCs were eluted with 2 mL 80% (v/v) methanol. The eluate were analyzed by Aglient HPLC 1100 system equipped with ODS (Cosmosil 5C18-AR, column 250 mm × 4.6 mm, Japan) kept at 40 °C. The mobile phases were composed of Milli-Q water containing 0.05% (v/v) trifluoracetic acid and HPLC quality methanol, which were blended at a rate of 45:55 over 25 min. The flow rate was 1 mL/min. The eluent was passed through a variable wavelength detector (VWD) operated at 238 nm and calculated against a standard curve with MC-LR and MC-RR (Sigma-Aldrich, USA).

### 3.3. Isolation of Bacterial Strain EMS with the Activity of MCs Degradation

A sludge sample was taken from the piled cyanobacteria fished out from Lake Taihu located in Wuxi, China. Five g of the sample was suspended in 50 mL sterilized water and shaken at 120 rev/min for 1 h at 30 °C. After standing for 30 min, 5 mL of the supernatant were inoculated into 50 mL Enrich Bacteria Broth (EBB) medium mixed with 200 mL crude MC extract. EBB was composed of 1.0 g/L MgSO_4_·7H_2_O, 0.5 g/L KH_2_PO_4_, 4.0 g/L K_2_HPO_4_, 1.0 g/L NaCl, 20 mg/L CaCl_2_, 5 mg/L FeSO_4_, 5 mg/L ZnCl_2_, 5 mg/L MnCl_2_·4H_2_O, 0.5 mg/L CuCl_2_, 2.0 g/L glucose, and 0.15 g/L yeast extract. After shaking at 120 rev/min for 5 days at 30 °C, 50 mL of the resulting solution were inoculated again into EBB containing crude MC extracts for subculture.

After five serial subcultures, the incubated medium was spread on Mineral Salts (MS) agar medium with 20% (v/v) of crude MCs extract. MS medium was composed of 1.0 g/L MgSO_4_·7H_2_O, 0.5 g/L KH_2_PO_4_, 4.0 g/L K_2_HPO_4_, 1.0 g/L NaCl, 20 mg/L CaCl_2_, 5 mg/L FeSO_4_, 5 mg/L ZnCl_2_, 5 mg/L MnCl_2_·4H_2_O, 0.5 mg/L CuCl_2_, and 20 g/L agar powder. Single colonies were transferred into aqueous MS medium with crude MC extract, respectively, shaking at 30 °C with the rate of 120 rev/min. The growth of bacteria was monitored by detecting the absorption of medium at 600 nm. The consumption of MC-LR and MC-RR by bacteria was quantified by detecting the concentration of MCs in the medium using HPLC. Among the tested colonies, one EMS strain showed the best activity in MC degradation.

### 3.4. Identification of the EMS Strain

Identification of the EMS strain was performed considering chemotaxonomic method and 16S ribosomal RNA gene sequence analysis [48]. Cell size was measured using microscopy with a haemacytometer (Nikon TE2000, Japan). Total DNA of EMS was isolated using UltraPureTM DNA extraction kit (SBS Gene, Shanghai, China). Bacterial gene encoding 16S rRNA was amplified by PCR using universal primers (Sense: 5’-AGAGTTTGATCCTGGCTCAG-3’; antisense: 5’-GGTTACCTT GTTACGACTT-3’) provided by Invitrogen Ltd., China. PCR was performed as follows: 95 °C for 3 min, 35 cycles at 94 °C for 30 s, 58 °C for 30 s, 68 °C for 1.5 min, and a final extension step at 68 °C for 7 min. The amplified nucleotide product was sequenced in Invitrogen Ltd., China. Similar sequences were identified using online BLAST in NCBI neulecotide database (http://blast.ncbi.nlm.nih.gov/Blast.cgi). A multiple alignment and a phylogenetic tree were obtained using CLUSTAL X 2.0 software [49] and MEGA 4 software [50]. The DNA sequence encoding 16S rRNA of strain EMS is available under GenBank accession number FJ712028.

### 3.5. Experiment Design of MCs Biodegradation by Strain EMS

Firstly, EMS was incubated with M9 medium containing crude MCs extract at various incubation temperatures, such as 4, 10, 20, 30, and 37 °C. Bacterial growth was detected by measuring the absorbance of the culture at 600 nm every 3 h. The results showed that EMS grew well at a temperature between 30 °C and 37 °C. Then EMS was incubated with M9 medium containing crude MC extract at 30 °C but with different pH values, such as 5.0, 6.0, 7.0, 8.0, and 9.0. The concentration of MC-LR and MC-RR in cultures and bacterial growth were detected by HPLC and absorbance measurement at 600 nm every 3 hours, respectively. At the end of the experiment, bacterial samples were taken for physical characterization and 16S rRNA gene sequence analysis to detect possible contamination by other bacteria.

### 3.6. PCR for mlrA and Sequence Analysis

EMS strain was cultured in M9 medium containing crude MCs extract for 48 hours. Cultures (1.3 mL) were centrifuged at 10,000 rev/min for five minutes. The precipitated bacteria were collected for genomic DNA extraction using an UltraPureTM DNA extraction kit (SBS Gene, Shanghai, China). The integrity of genomic DNA was detected in agarose gel (1%). This genomic DNA from EMS was employed as DNA template in the next PCR process.

The primers employed for the amplification of *mlrA* (accession number AF411068) fragment in this experiment were 5’-GACCCGATGTTCAAGATACT-3’ (sense) and 5’-TTAATCTTCATGCTGCTAGGAGC-3’ (antisense) [28]. PCR was performed as follows: 95 °C for 5 min, 35 cycles at 94 °C for 30 s, 52 °C for 30 s, 72 °C for 60 s, and a final extension step at 72 °C for 10 min. The size of the amplified PCR product (*mlrA-EMS*) was detected in agarose gel (1%) and the sequence was identified in Invitrogen Ltd., China. Identification of similar sequences and multialignment were performed by online BLAST searches from Genebank (http://www.ncbi.nlm.nih.gov/BLAST). The GeneBank accession number of *mlrA-EMS* is GU224277. Protein sequence of MlrA was predicted and analyzed by DNAStar software (DNASTAR, Inc., Wisconsin, USA). Analysis of hydrophilicity was performed by ExPASy ProtParam Tool (http://expasy.org/cgi-bin/protparam). Analysis of alpha helix and transmembrane region were performed by TMHMM tool (http://www.cbs.dtu.dk/services/TMHMM-2.0) and TEMpred tool (http://www.ch.embnet.org/software/TMPRED_form.html). Analysis of subcellular location was performed by POSRT II Prediction tool (http://psort.ims.u-tokyo.ac.jp/form2.html).

## 4. Conclusions

From our results, an isolated EMS bacterial strain showed high efficiency of microcystin-LR and RR degradation, which contributed to the bioremediation microcystin pollution due to toxic cyanobacterial blooms. A *mlrA* gene involved in microcystin biodegradation was detected in EMS. The analysis of *mlrA-EMS* was expected to promote the characterization of the unknown protein MlrA that might belong to a new protease family having unique functions.

## Figures and Tables

**Table 1.** Similarities of *mlrA-EMS* with other reported *mlrA* genes in DNA database.

**Bacterial strains**	**GeneBank accession number in NCBI**	**Similarity with *mlrA-EMS***	**Reference**
*Sphingopyxis* sp. C-1	AB468058	98%	Direct submisson
*Sphingopyxis* sp. LH21	DQ112243	98%	Direct submisson
*Sphingomonas* sp. ACM-3962	AF411068	92%	[34]
*Sphingomonas* sp. MD-1	AB114202	91%	[28]
*Sphingomonas* sp. Y2	AB114203	83%	[28]
